# Supplementary material for: Development of Taccalonolide AJ-Hydroxypropyl-β-Cyclodextrin Inclusion Complexes for Treatment of Clear Cell Renal-Cell Carcinoma
Source: Molecules. 2020 Nov 27;25(23):5586. doi: 10.3390/molecules25235586 (PMC7731059; doi:10.3390/molecules25235586)
Supplement: Supplementary file 1 [file molecules-25-05586-s001.pdf]

Supplementary

Development of taccalonolide AJ-hydroxypropyl-β-cyclodextrin inclusion complexes for treatment of clear cell renal-cell carcinoma

Jing Han[1,†], Siwang Zhang[1,†], Junxin Niu[2,†], Chunli Zhang[1], Weichen Dai[1], Yuanyuan Wu[1,*], Lihong Hu[1,2,*]

1 Jiangsu Key Laboratory for Functional Substance of Chinese Medicine, Jiangsu Collaborative Innovation Center of Chinese Medicinal Resources Industrialization, State Key Laboratory Cultivation Base for TCM Quality and Efficacy, Jiangsu Key Laboratory for Pharmacology and Safety Evaluation of Chinese Materia Medical, Nanjing University of Chinese Medicine, Nanjing, 210023, PR China. Jiangniao172@163.com (J. H), 15951878760@163.com (S.-W. Z), 122758476@qq.com (C.-L. Z), 753518122@qq.com (W.-C. D), ywu@njucm.edu.cn (Y.-Y. W), lhhu@njucm.edu.cn (L.-H .H).
2 State Key Laboratory of Drug Research, Shanghai Institute of Materia Medical, Chinese Academy of Sciences, 501 Haike Road, Shanghai 201203, People's Republic of China; 1339703266@qq.com (J.-X. N)
* Correspondence: lhhu@njucm.edu.cn, ywu@njucm.edu.cn, Nanjing University of Chinese Medicine, No.138, Xianlin Road, Nanjing, 210023, China; Tel.: +86-25-85811353; Fax: +86-25-85811355.

Table of Contents

Materials and Methods..........3

Figure S1. ^1H NMR spectrum of AJ in MeOD..........4

Figure S2. ^1H NMR spectrum of HP-β-CD in D_2O..........5

Figure S3. ^1H NMR spectrum of AJ+HP-β-CD in MeOD..........6

Figure S4. ^1HNMR spectrum of AJ-HP-β-CD in D_2O..........7

Figure S5. The tissue distribution of AJ-HP-β-CD and AJ *in vivo*..........8

Figure S6. The standard curve of AJ..........9

Figure S7. The plasma pharmacodynamics of AJ-HP-β-CD and AJ..........10

Figure S8. The bodyweight for acute toxicity test after AJ-HP-β-CD injection on rat..........11

STable 1. The Results of solubility studies of HP-α-CD, HP-β-CD, HP-γ-CD and SBE-β-CD..........12

STable 2. The LD_{50} and MTD data of AJ-HP-β-CD..........13

Materials and Methods

Acute toxicity study

Twenty-four SD rats weighing 180 ± 20 g were randomly assigned into four groups (n = 6, half male and female) for the acute toxicity study, which were the control and three treatment groups of AJ-HP-β-CD at two fixed doses (5, 10, and 20 mg/kg of body weight), respectively. AJ-HP-β-CD was diluted in saline, and the control group received the same volume of saline. All the treatments were injected via tail intravenous administration on the first day only. Body weights and survival were monitored on a daily basis between 2 weeks.

Plasma pharmacokinetics study

Twelve male SD rats were randomly assigned into two groups to carry out the plasma pharmacokinetics test *in vivo*. Fasting before administration, one group was administered AJ by gavage (2 mg/kg), while the other was given AJ-HP-β-CD by intravenous injection (a dose-content 2 mg/kg AJ). Blood samples were collected from the orbital sinus at a specified time.

Tissue distribution test

AJ (2 mg/kg) was given by gavage, AJ-HP-β-CD (2 mg/kg AJ) was delivered by intravenous injection; the rats (6 rats per time point) were euthanized at the time points of 5, 10, 30, 60 and 100 min, and the kidneys were immediately excised. The harvested kidneys were rinsed carefully with saline solution and then homogenized with a tissue homogenizer after an appropriate amount of ultrapure water (1 mL/0.1 g tissue) was added, and the obtained homogenates were pre-treated and assayed by ELSD-HPLC.

Figure S1. ^{1}H NMR spectrum of AJ in MeOD

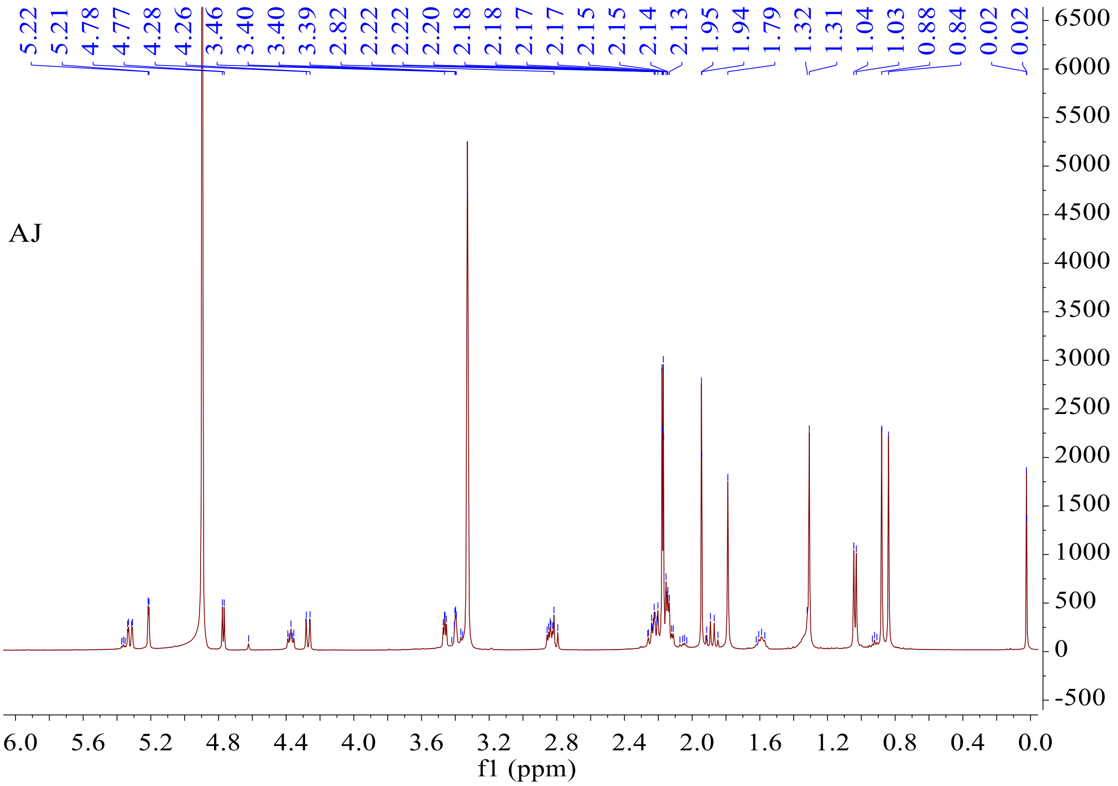

Figure S2 ^{1}H NMR spectrum of HP-β-CD in D_2O

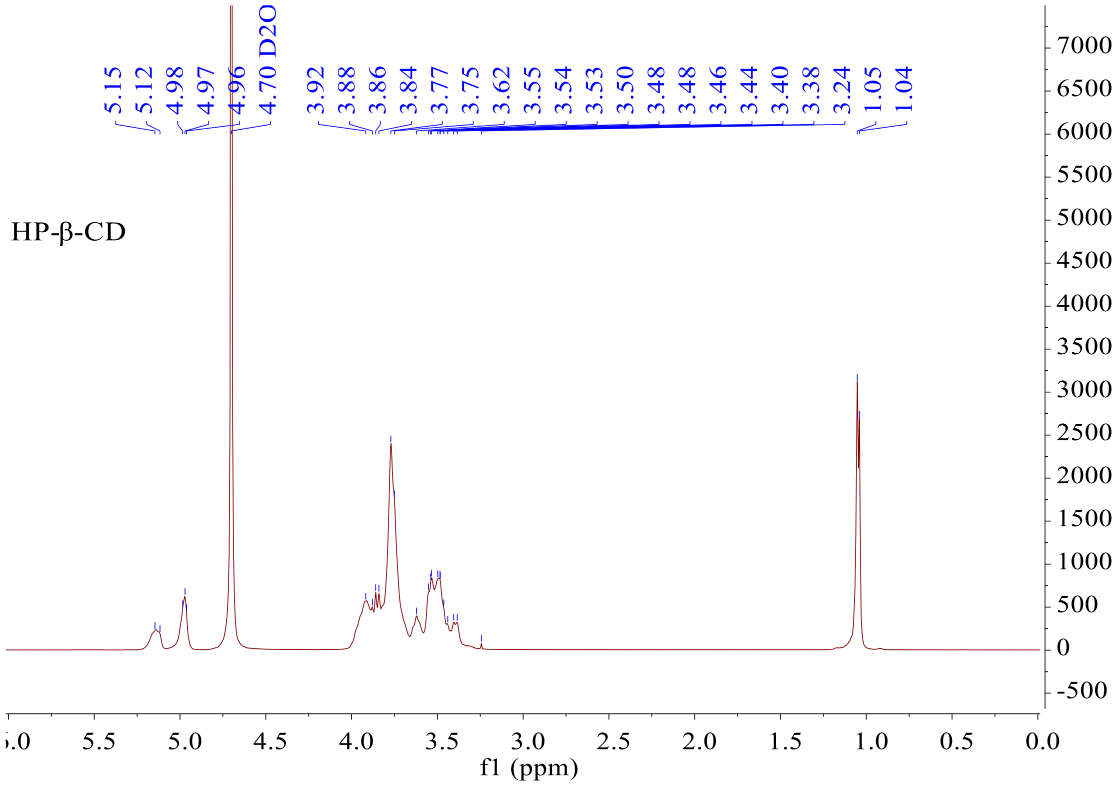

Figure S3 ^{1}H NMR spectrum of AJ + HP-β-CD in MeOD

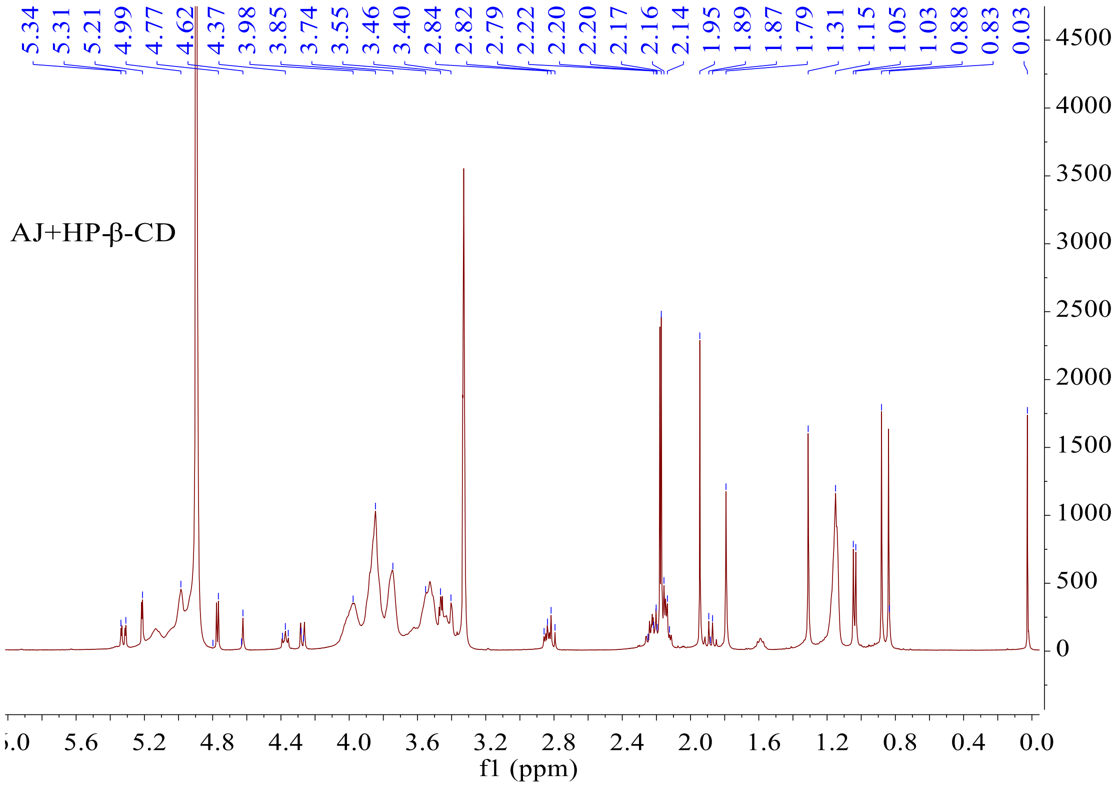

Figure S4. ^1HNMR spectrum of AJ-HP-β-CD in D_2O

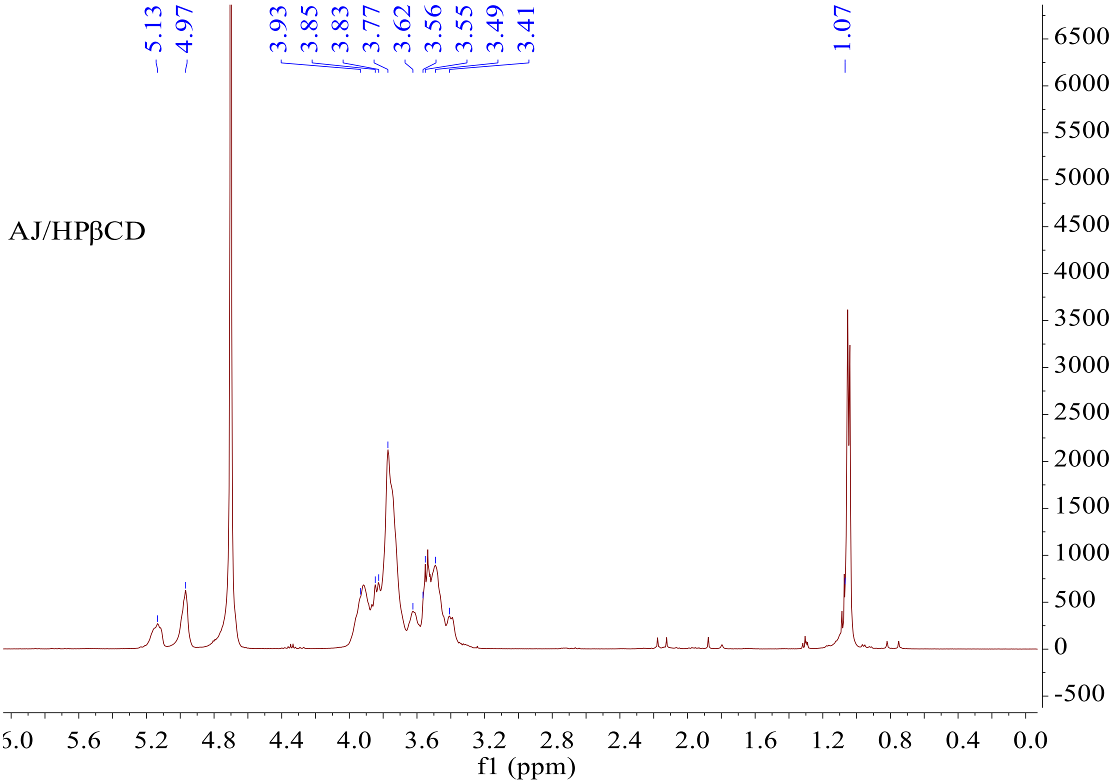

Figure S5. The tissue distribution of AJ-HP-β-CD and AJ *in vivo*

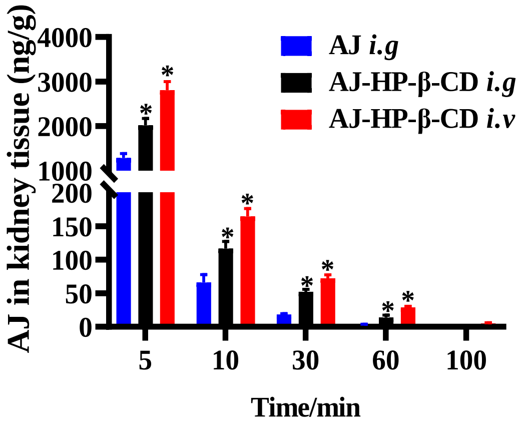

Figure S6. The standard curve of AJ

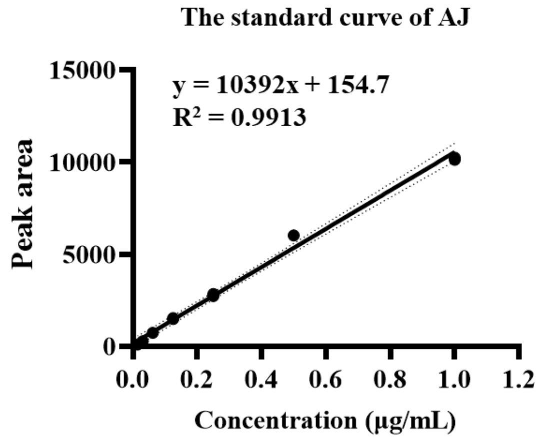

Figure S7. The plasma pharmacodynamics of AJ-HP-β-CD and AJ

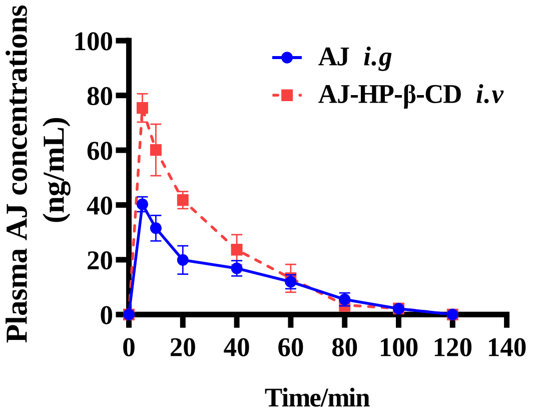

Figure S8. The changes of bodyweight for acute toxicity test after AJ-HP-β-CD injection on rat

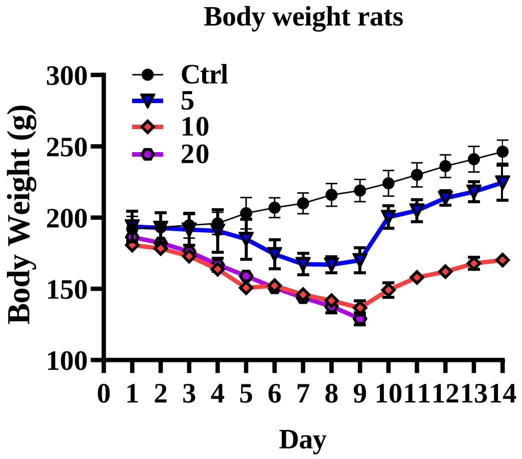

STable 1. Results of solubility studies of HP-α-CD, HP-β-CD, HP-γ-CD and SBE-β-CD

NO	Type of Cyclodextrin	solvent	solubility
1	HP-α-CD	alcohol	no
2	HP-α-CD	DMF	yes
3	HP-β-CD	alcohol	yes
4	HP-γ-CD	alcohol	yes
5	SBE-β-CD	alcohol	no
6	SBE-β-CD	water	no

STable 2. The LD_{50} and MTD data of AJ-HP-β-CD

Group	Animals	Dose (mg/kg)	Injection volume (mL)	Mortality rate (%)
Control	10	0	0.4	0
AJ-HP-β-CD-1	10	2.06	0.4	0
AJ-HP-β-CD-2	10	4.74	0.4	0
AJ-HP-β-CD-3	10	10.71	0.4	0
AJ-HP-β-CD-4	10	25.35	0.4	20
AJ-HP-β-CD-5	10	60	0.4	50

LD_{50} = 60 mg/Kg

MTD = 10.71 mg/Kg
